# Wi-Filter: WiFi-Assisted Frame Filtering on the Edge for Scalable and Resource-Efficient Video Analytics

**DOI:** 10.3390/s25030701

**Published:** 2025-01-24

**Authors:** Lawrence Lubwama, Jungik Jang, Jisung Pyo, Joon Yoo, Jaehyuk Choi

**Affiliations:** School of Computing, Gachon University, 1342 Seongnam-daero, Sujeong-gu, Seongnam-si 13120, Republic of Korea; lawrencejews@gmail.com (L.L.); jji4449@gachon.ac.kr (J.J.); p990301@gachon.ac.kr (J.P.)

**Keywords:** Wi-Fi sensing, channel state information, video frame filtering, 1D CNN, edge computing

## Abstract

With the growing prevalence of large-scale intelligent surveillance camera systems, the burden on real-time video analytics pipelines has significantly increased due to continuous video transmission from numerous cameras. To mitigate this strain, recent approaches focus on filtering irrelevant video frames early in the pipeline, at the camera or edge device level. In this paper, we propose Wi-Filter, an innovative filtering method that leverages Wi-Fi signals from wireless edge devices, such as Wi-Fi-enabled cameras, to optimize filtering decisions dynamically. Wi-Filter utilizes channel state information (CSI) readily available from these wireless cameras to detect human motion within the field of view, adjusting the filtering threshold accordingly. The motion-sensing models in Wi-Filter (Wi-Fi assisted Filter) are trained using a self-supervised approach, where CSI data are automatically annotated via synchronized camera feeds. We demonstrate the effectiveness of Wi-Filter through real-world experiments and prototype implementation. Wi-Filter achieves motion detection accuracy exceeding 97.2% and reduces false positive rates by up to 60% while maintaining a high detection rate, even in challenging environments, showing its potential to enhance the efficiency of video analytics pipelines.

## 1. Introduction

In recent years, intelligent video analytics systems have become increasingly prevalent across various fields, such as the Internet of Things (IoT), public safety, transportation, and numerous industrial applications. These systems play a vital role in a wide range of scenarios, from monitoring buildings, airports, train stations, schools, and universities, to managing urban and highway traffic. They are designed to detect critical events, such as car accidents or crowd surges, and promptly issue relevant alerts [1,2].

One of the major challenges faced by these systems is achieving real-time processing to deliver timely and actionable insights across diverse activities and scenarios. Typically, video analytics servers runs on centralized servers located in monitoring centers or on cloud platforms, which offer powerful processing capabilities and virtually unlimited storage. Despite these advantages, traditional centralized or cloud-based systems often struggle to maintain real-time responsiveness across a large number of connected devices. This limitation arises from the continuous transmission of high-resolution video streams from multiple cameras, which imposes significant computational and network burdens on centralized systems. The high processing demands, coupled with substantial network bandwidth requirements, further intensifies these challenges, hindering efficient and scalable system performance.

Edge computing has emerged as a promising solution to overcome these challenges [2,3,4]. By shifting computation from centralized data centers to end-user devices and local networks, edge computing extends the capabilities of cloud architecture to the network’s edge. This paradigm enables the deployment of low-latency, innovative services that meet the increasing demand for real-time responsiveness. However, edge computing also faces challenges, particularly when high-resolution data are continuously streamed from numerous cameras, which results in significant computational and network load on the edge infrastructure. This emphasizes the importance of developing efficient and scalable solutions to enhance the performance of video analytics systems at the edge.

One effective approach to mitigate these challenges is frame filtering at the early stages of the video analytics pipeline—at the camera and/or edge devices—which assesses frame confidence and decides whether to transmit frames for further processing. Several filtering techniques have been developed to tackle this issue, generally categorized into three main models: (i) compressed object detection models [5,6,7], (ii) binary classification models [8], and (iii) pixel-level frame differencing methods [9].

Despite such efforts, the capabilities of the existing solutions are still highly limited. These approaches commonly rely on preconfigured static thresholds [5,9,10] due to their simplicity and efficiency. However, optimal filtering thresholds are highly dependent on video content, which can vary dynamically, reducing their effectiveness. Additionally, object recognition in low-light environments or under occlusion remains a persistent challenge in camera-based approaches.

In this paper, we demonstrate how Wi-Fi-based activity recognition opens up a novel opportunity for frame filtering, overcoming the limitations of static thresholds used in previous approaches. Our key innovation lies in detecting motion within a camera’s field of view (FoV) by leveraging human-induced variations in the Wi-Fi channel between the camera and a Wi-Fi access point (AP). This enables dynamic adjustments in filtering decisions based on real-time motion detection.

We introduce Wi-Filter, a novel filtering method aimed at maximizing the benefits of edge filtering in video analytics pipelines for Wi-Fi-enabled cameras. Wi-Filter utilizes channel state information (CSI), inherently available in wireless cameras, to detect human motion in the FoV and dynamically adjust the filtering threshold accordingly.

Our approach uses a data-driven methodology to capture the unique temporal and spectral features of Wi-Fi CSI through lightweight learning models, enabling effective human motion detection with minimal computational overhead. To our knowledge, Wi-Filter is the first frame-filtering technique to employ Wi-Fi-based activity recognition. We validate the effectiveness of Wi-Filter through a prototype implementation, supported by extensive experimental evaluations that showcase its potential to significantly improve video analytics systems.

The main contributions of this paper are summarized as follows:

- Introduction of Wi-Filter: We propose Wi-Filter, a novel filtering framework that leverages Wi-Fi signals from wireless edge devices, such as Wi-Fi-enabled cameras, to dynamically adjust the filtering threshold. To the best of our knowledge, this is the first approach that utilizes Wi-Fi-based activity recognition to enhance frame filtering mechanisms.
- On-device CSI Data Processing with Lightweight 1D CNN: Our proposed framework employs a one-dimensional convolutional neural network (1D CNN) instead of the commonly used 2D CNN models in Wi-Fi sensing for processing run-time collected CSI data. This choice allows the filtering module to operate on-device with minimal computational overhead, making it a more practical solution for resource-constrained environments.
- Prototype Implementation and Evaluation: We implemented a prototype of Wi-Filter using low-capacity edge devices and a GPU-enabled edge server. Testbed deployments demonstrated that our system achieved the same frame analysis accuracy while significantly reducing the GPU and CPU load of the cloud server, as well as network traffic.
- Comprehensive Summary of Recent Work: We also provide a comprehensive summary of intelligent video analytics systems, highlighting relevant research and existing methods to contextualize the importance and contribution of our proposed solution.

The remainder of this paper is organized as follows. Section 2 provides a summary of the work related to our approach. Section 3 presents the background and motivation behind this study. Section 4 offers an overview of Wi-Filter and elaborates on its system design. Section 5 presents the evaluation results, demonstrating the applicability of Wi-Filter. Finally, in Section 6, we discuss potential future extensions and conclude the paper.

## 2. Related Work

This section provides a comprehensive overview of research efforts in scalable video analytics at the edge, focusing on three key areas: (i) efficient video analytics at the edge, (ii) filtering techniques developed to address the challenges discussed in this paper, and (iii) WiFi-based sensing technologies, which have emerged as a promising tool for non-invasive detection and analysis.

### 2.1. Efficient Video Analytics at the Edge

In a recent study [11], an idea to send the video stream in low resolution, but recover the high-resolution frames from the low-resolution stream via a super-resolution procedure tailored for the actual analytics tasks, has been proposed. This paper presented an edge-to-cloud framework for advanced vision analytics, CloudSeg, which achieves low latency and high inference accuracy despite limited network bandwidth. CloudSeg sends video streams in low resolution and uses a cloud-side super-resolution process to recover high-resolution frames, effectively reducing bandwidth requirements while maintaining accuracy. The paper [12] presented a solution to improve real-time object detection in edge systems using multi-model and multi-device detection parallelism. By analyzing performance bottlenecks and optimizing detection through parallel processing, the study demonstrated significant improvements in frame-per-second (FPS) processing rates, thereby achieving near real-time detection performance on heterogeneous edge devices for efficient video analytics. In [13], the concept of “VAP performance clarity” was introduced to address the challenges in evaluating video analytics pipelines (VAPs), which are used in edge video analytics to balance inference accuracy and resource cost. The authors presented “Yoda”, the first benchmark designed to characterize the interaction between VAPs and video content, providing a precise performance clarity profile to define the accuracy vs. cost trade-off. The accuracy–latency trade-off for real-time deep video analytics at the edge is investigated in [14], focusing on YOLO-based object detection and WebRTC-based video streaming. It proposes a mechanism to adapt video streaming settings, such as bitrate and resolution, to enhance accuracy while maintaining low latency, and demonstrates its efficiency in finding optimal configurations through simulations. The paper [15] introduced an approach to enhance the reliability of deep-learning-based object detection on resource-constrained edge devices by transforming a state-of-the-art detection algorithm into a task optimization problem. Using a semi-partitioned rate-monotonic scheduling algorithm, the proposed method improved real-time video inference performance and enhanced system reliability and detection accuracy. A recent study in [16] proposed a solution for compressing video content live-streamed from devices to the edge, while maintaining accuracy and timeliness in video analytics. The proposed solution utilized offline profiling and online adaptation to optimize on-device processing for video compression, showing superior performance compared to state-of-the-art methods across various object detection tasks and datasets. A joint video query scheduling and resource allocation approach is introduced in [17] for low-latency, accuracy-guaranteed video analytics in edge-based systems. The problem is formulated as a Markov decision process to handle uncertainties in resource demands and scheduling, and an edge-coordinated reinforcement learning algorithm is proposed to adaptively manage query scheduling and resource allocation. Simulation results demonstrated that the proposed method significantly improved latency and accuracy. A recent study [18] proposed a novel cascade architecture designed to address the limitations of existing retrospective analytics systems by splitting the cascade computation between the compressed and pixel domains. The proposed solution, CoVA, handled both temporal and spatial queries, cascading the analysis into three stages, where the first two operate in the compressed domain and the final stage in the pixel domain. By selectively decoding a minimal set of frames, CoVA alleviates the decoding bottleneck and significantly improves performance.

### 2.2. Filtration for Analytics at the Edge

Related to the second category, several filtering strategies have been proposed to reduce computing overhead and minimize edge-to-cloud data transfer in large-scale video analytics systems. These strategies can be categorized into three main models: compressed object detection models [5,6,7], binary classification models [8], and pixel-level frame differencing methods [9].

One of the earliest and most relevant studies related to video filtering for video analytic systems, Glimpse was introduced as a continuous, real-time object recognition system for camera-equipped mobile devices, proposed in [9]. To mitigate accuracy loss due to server latency, Glimpse maintained an active cache of video frames on the mobile device and employs trigger frames to minimize network bandwidth usage. Experiments with Android smartphones and Google Glass demonstrated that Glimpse significantly improves recognition precision, achieving very high accuracy. In [6], the authors presented Focus, a system aimed at providing low-cost and low-latency querying for large video datasets recorded for traffic control and surveillance. Focus splits query processing between ingest time and query time by using lightweight CNN classifiers to create an approximate index at ingest time, and compensates for reduced accuracy by employing more expensive CNNs during query time. The experimental results demonstrated that Focus achieved significantly lower query latency compared to state-of-the-art video querying systems. In [8], the authors introduced FilterForward, an edge-to-cloud system aimed at managing large-scale video camera deployments by utilizing lightweight edge filters to backhaul only relevant video frames. FilterForward employed fast “microclassifiers” to share computation and detect multiple events on resource-constrained edge nodes, minimizing the transmission of full video streams. Evaluations on real-world datasets demonstrated that FilterForward significantly enhances computational efficiency and event detection accuracy while reducing network bandwidth usage. The study [10] addressed the high resource demands of real-time video analytics pipelines by exploring on-camera filtering, shifting the filtering process to the start of the pipeline. Existing approaches have often relied on neural networks running on edge or backend servers, which can be costly. However, commodity cameras lack the resources to run such intensive operations and are typically limited to frame differencing based on low-level features, which can compromise query accuracy if used improperly. To tackle this issue, the authors propose Reducto, a system that dynamically adapts filtering decisions according to the correlations among feature type, filtering threshold, query accuracy, and video content. The experimental results show that Reducto can filter out 51–97% of frames while maintaining the desired accuracy. In [5], the authors have proposed an edge computing-based system for scalable video analytics that reduces data transfer between surveillance cameras and cloud servers by leveraging information from the encoded bitstream. The proposed approach focuses on minimizing processing complexity by filtering non-motion frames and performing object tracking at the edge. Evaluation results demonstrated that the system, implemented using low-capacity edge devices and a GPU-enabled server, achieved the same frame analysis accuracy while significantly reducing the cloud server’s GPU, CPU load, and network traffic. In a recent study [7], researchers proposed the cross-video filtration (CVF) framework to enhance the efficiency of deep neural network (DNN)-based video analytics in edge applications. Recognizing that only a small portion of camera streams typically contain objects of interest, CVF aims to reduce unnecessary processing of all frames with resource-intensive DNNs. The framework employs compressed-domain data, lightweight classification models, and a prioritization algorithm to filter frames across multiple streams based on content and resource constraints. The experimental results demonstrate that CVF effectively reduces response times and improves system efficiency in multi-camera environments.

### 2.3. WiFi-Based Sensing Technologies

WiFi-based sensing technology has recently gained significant attention as a contactless method for human presence detection across various domains, including smart homes, healthcare, and security systems. The attention-enhanced deep learning for presence detection (ALPD) system [19] demonstrates high detection accuracy by leveraging CSI data. Through attention mechanisms, ALPD identifies important CSI subcarriers and employs bidirectional LSTM networks to learn temporal dependencies. This approach achieves superior performance in both static and dynamic scenarios, outperforming traditional methods even in multi-room environments, thereby opening new technical possibilities. Similarly, the time-selective condition dual feature extraction recurrent network (TCD-FERN) system [20] addresses the challenge of multi-room presence detection by separating CSI data into dynamic and static features. TCD-FERN achieves robust performance in both LoS (line-of-sight) and NLoS (non-line-of-sight) settings. By incorporating a time selection mechanism and a voting-based method, the system enhances detection accuracy to over 97%, demonstrating cost-effectiveness by utilizing only a limited number of APs. Another study [21] applies signal processing techniques, such as Fourier transform and wavelet transform, to CSI data, followed by analysis using neural networks. By integrating a preprocessing pipeline combining low-pass filters and wavelet transforms to reduce noise and extract salient features, the study achieves an impressive detection accuracy exceeding 99%, even in complex indoor environments. Beyond presence detection, WiFi-based sensing has shown potential for more complex tasks, such as multi-class activity recognition. By increasing model complexity and integrating advanced feature extraction with deep learning, these systems can classify diverse human activities. For instance, the authors of [22] developed a human activity recognition (HAR) system utilizing MIMO antennas and an LSTM-based deep learning model, achieving a detection accuracy of 97.5% across seven activities, including those conducted through walls. The system’s reliability in NLoS environments demonstrates the effective use of multipath propagation. Further extending the capabilities of WiFi-based HAR, the AFE-MatNet framework [23] achieves high detection accuracy without retraining in dynamic environments. By filtering irrelevant data and enhancing activity-specific features, AFE-MatNet maintains accuracy above 90% across diverse settings, highlighting its potential for environment-independent applications.

These studies underscore the transformative potential of WiFi-based sensing technologies, evolving from simple presence detection to sophisticated applications, such as complex human activity recognition, thereby paving the way for broader adoption in various real-world scenarios.

## 3. Motivation

Wi-Filter addresses the limitation of previous approaches that rely on static or predetermined thresholds for frame filtering. To motivate the need for a more adaptive solution, we first conducted experiments to evaluate the performance of a widely used binary classification approach [8] in controlled environments.

**Figure 1:**
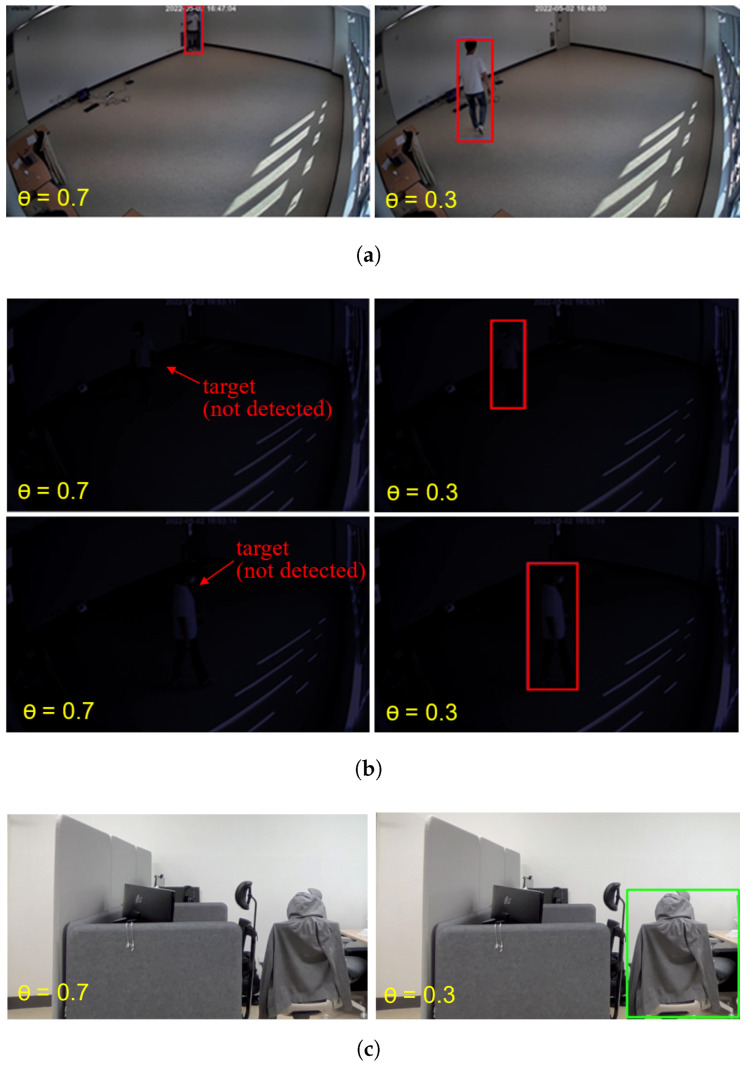
Limitations of static, predefined threshold selection. (**a**) In bright conditions, YOLOv4 accurately identified the target regardless of the threshold value. (**b**) Under low-light conditions, YOLOv4 struggled with detection failures at a high threshold value (θ=0.7) but correctly identified the target with a lower threshold (θ=0.3). (**c**) A very low threshold may lead to false positives, such as misidentifying a chair as a human, resulting in unnecessary video frame forwarding.

Figure 1 presents the motion detection results obtained using a simple binary classification method based on YOLO [24] under two different lighting conditions, (a) bright environments and (b) dark environments, with two different thresholds, θ=0.7 and θ=0.3. With θ=0.7, human targets were accurately identified under bright conditions; however, no motion was detected under dark conditions, leading to the inaccurate filtering of frames despite the presence of the target.

On the other hand, with θ=0.3, the motion detection detector, i.e., the YOLO-based binary classifier, successfully detected human motion in both bright and dark conditions. However, Figure 1c indicates that using a lower threshold can easily lead to false detection and will pass frames that do not contain relevant information. In summary, filtering accuracy may be compromised when the threshold is set too high, whereas the filtering effectiveness is diminished when the threshold is too low. Given the inherent variability of video content, determining an optimal threshold is challenging, which highlights the need for a more adaptive and dynamic filtering approach.

**Figure 2:**
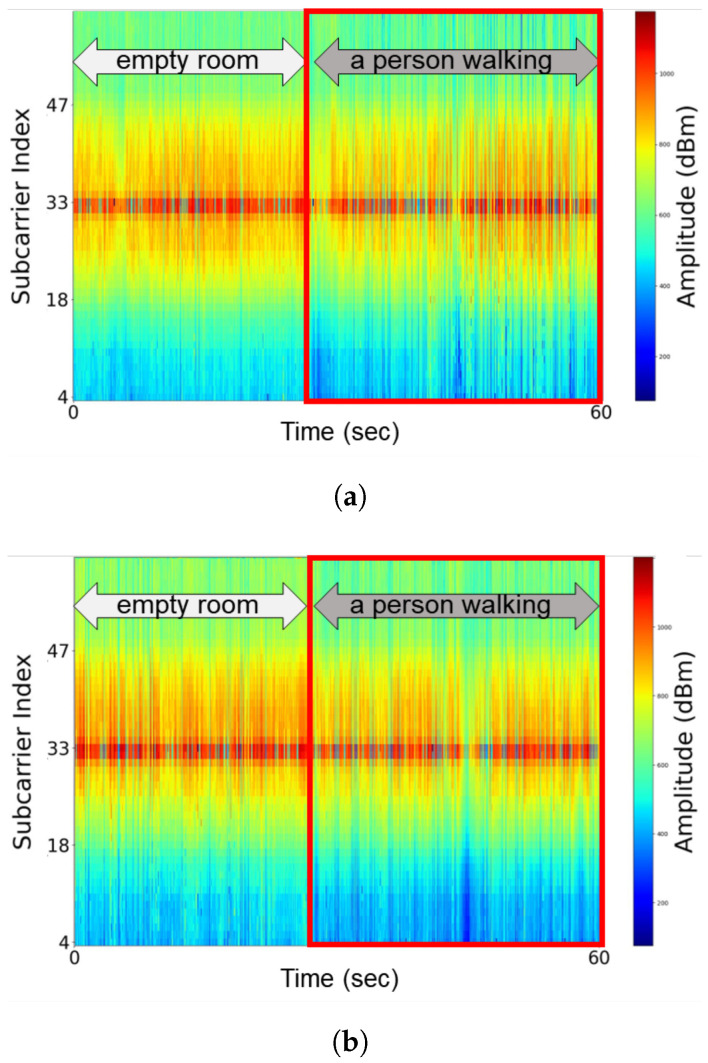
CSI spectrogram obtained for two different states, no person in the room and one person walking in the room, under (**a**) bright and (**b**) dark conditions.

To overcome this challenge, we recognized the fact that the propagation of signals through a wireless channel is affected by the composition of the surrounding environment and the presence of obstacles. The physical changes in wireless channels can be accurately captured through CSI analysis based on the opportunistic use of Wi-Fi signals [25]. Figure 2 presents a spectrogram of Wi-Fi CSI amplitudes for two different activities, no human activity and one person walking in the target area, under (a) bright and (b) dark conditions. It can be seen that the patterns in the CSI data differ significantly depending on whether there is human activity in the target area regardless of the lighting condition, that is, of whether it is a dark or bright environment. Wi-Filter harnesses patterns in the Wi-Fi signal to dynamically adjust thresholds to maximize filtering benefits and handle inaccurate frame filtering. In particular, when no motion is detected as a result of Wi-Fi sensing, a high threshold is selected to trigger frame filtering, and, conversely, when motion is detected, a low threshold is selected to suppress inaccurate frame filtering.

## 4. Wi-Filter System Design

In this section, we introduce the system design of Wi-Filter, a novel efficient frame-filtering method used for camera-based video streams.

### 4.1. Overview

**Figure 3:**
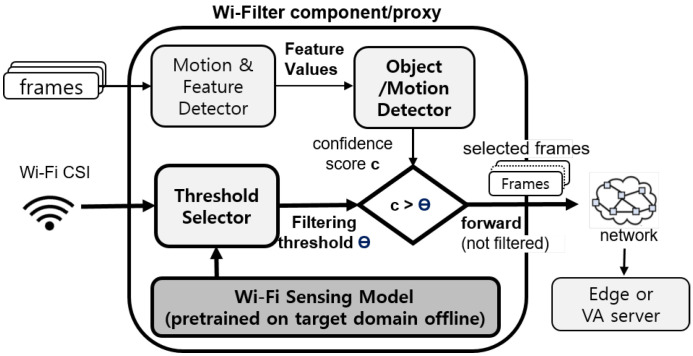
Wi-Filter architecture.

An overview of the Wi-Filter architecture is illustrated in Figure 3. Wi-Filter can be implemented as an on-device module on the wireless camera itself or deployed on edge devices as a proxy component, where the camera streams the frames to the Wi-Filter proxy located at the edge via a Wi-Fi connection.

Wi-Filter consists of two stages: (i) the data collection stage and (ii) the execution stage. In the first stage, CSI data collection and automatic labeling are performed for pre-training a lightweight Wi-Fi-based motion sensing model. In this step, the motion detector (for example, YOLOv4) performs frame filtering in the conventional way using a predefined (for example, θ=0.7) threshold. When the training of the Wi-Fi sensing model is completed, the execution stage begins. In the execution stage, the trained Wi-Fi sensing model acts as a threshold selector and derives the filtering threshold θ using real-time measured CSI data as the input. The θ value overrides the predefined threshold of the motion detector to dynamically perform frame filtering.

### 4.2. Data Collection and Auto-Labeling Stage

Threshold selector is a lightweight binary classifier that takes the measured Wi-Fi CSI samples as the input and determines the presence or absence of motion in the target domain where the camera is installed. Binary class labels are required for the measured CSI data for model training. We first present a brief background of the CSI.

The channel response of a wireless communication link depends heavily on the environment between a pair of transmitter (TX) and receiver (RX) antennas [26], and CSI can accurately reflect the physical changes in the wireless channel. In orthogonal frequency division multiplexing (OFDM) communication systems, it is assumed that the general model of CSI can be represented as follows:

(1) y=Hx+noise,

where **y** is the vector signal received, **x** is the signal vector transmitted, noise represents the Gaussian noise, and **H** denotes the channel gain information. Multiple-input multiple-output (MIMO) and OFDM systems have provided much richer information for multiple OFDM sub-carriers for each pair of TX-RX antennas to enable a sophisticated analysis of the packets transmitted. The transmitter antenna *i* provides a set of values $H_{i,r}$ to the receiver *r*, where $H_{i,j}$ represents a vector containing complex pairs captured for each sub-carrier represented as illustrated:

(2) $$h\left(f;t\right)=\sum_{i=1}^{N}a_{i}\left(t\right)e^{-j2\pi f\gamma_{i}\left(t\right)},$$

where $a_{i}\left(t\right)$ is the power attenuation, $\gamma_{i}$ is the propagation delay, *N* represents the number of multi-path components, and *f* is the carrier frequency. Several CSI extraction tools have been published for various Wi-Fi chipsets over the last decade [25,27]. We use the Nexmon CSI Extractor [26] to extract the CSI data.

**Figure 4:**
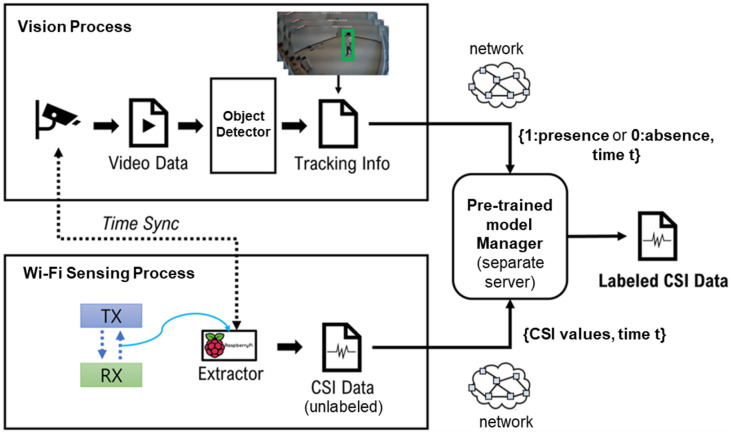
CSI auto-labeling architecture for Wi-Filter.

To train our neural network models for Wi-Fi-based motion sensing, we leverage a self-supervised approach to collect a large labeled CSI dataset. Figure 4 illustrates an auto-labeling system that collects Wi-Fi CSI data and performs automatic labeling for the Wi-Filter threshold selector. The detection results obtained from an object detector such as YOLOv4 for each frame are recorded along with the time information, and are periodically transmitted to a server together with the measured CSI samples.

The server performs time synchronization between the video and Wi-Fi CSI data as follows. First, the period for continuously recognizing an object using the result of the object detector is derived as $T_{k}$, containing the frame start time $t_{k}^{s}$ and end time $t_{k}^{e}$, and added to a set $T=\{T_{1},T_{2},\ldots \}$. Thereafter, the annotation is performed by determining whether each unlabeled CSI sample *CSI-x* is measured simultaneously with $\exists T_{k}\in T$ based on the following criteria:

Label of the sample $CSI_{x}$ is set to $\left\{\begin{array}{c} 1,\;\mathrm{if}\;t_{k}^{s}\leq t_{\mathrm{CSI-}x}\leq t_{k}^{s},\\ 0,\;\mathrm{otherwise}. \end{array}\right.$

In particular, when the timestamp of a CSI sample is in any $T_{k}=\left[t_{k}^{s},t_{k}^{e}\right]\in T$, it is annotated as 1, otherwise it is annotated as 0.

### 4.3. Run-Time Stage

In recent years, numerous deep learning (DL) architectures have been explored in the literature for Wi-Fi-based human sensing, including multilayer perceptron (MLP), convolutional neural networks (CNN), simple recurrent neural networks (SRNN), long short-term memory (LSTM), bidirectional LSTM (BiLSTM), gated recurrent unit (GRU), residual network (ResNet), autoencoders, transformers, and hybrid models [28] (refer to [28] for further details).

Unlike these sophisticated models for Wi-Fi based gesture and activity recognition, Wi-Filter aims to design a lightweight binary classifier, enabling on-device operation even in resource-constrained systems such as wireless cameras. Accordingly, we choose to apply lightweight models including long short-term memory (LSTM) and one-dimensional convolutional neural networks (1D CNN), which are widely used for learning time-series data, and random forest to Wi-Filter by considering the size of each sample and training data set. We will describe the experiments conducted to test the efficacy of these three different models in Section 5.

**Figure 5:**
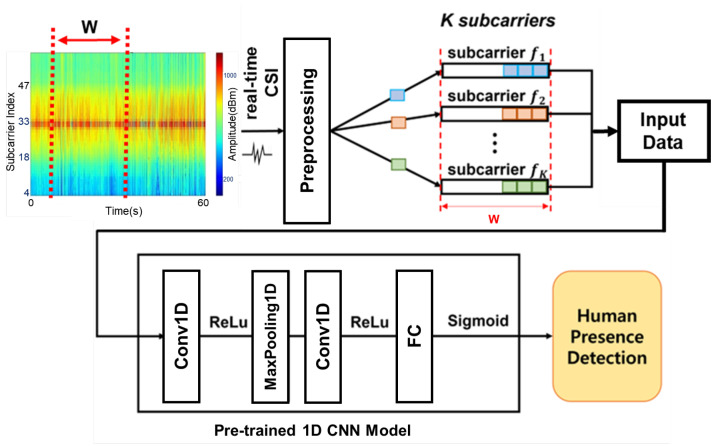
Overview of the real-time human presence detection process and lightweight CNN-based binary classifier architecture for the threshold selector.

Figure 5 illustrates the real-time human presence detection process for the 1D CNN model of Wi-Filter’s threshold selector. First, the raw CSI samples acquired in real-time are converted into amplitude values and normalized. Thereafter, the input data are formed by combining the data of the window size *W* for each *K* sub-carrier selected in the pre-training. The detailed data collection and preprocessing procedure is as follows. Note that this procedure is also applied to the model’s pre-training process, as described in Section 4.2.

- **MAC Filtering and Feature Removal**: During the preprocessing stage, packets are filtered based on their MAC addresses to ensure only data relevant to the target devices are processed. Then, irrelevant features such as MAC addresses and timestamps are removed from the collected packets to focus on the CSI values and minimize the data size.
- **Subcarrier Selection**: Among the subcarriers, 12 subcarriers—including null subcarriers (used to protect the band and enable coexistence with other wireless technologies) and pilot subcarriers (used for Wi-Fi link control)—are excluded. This leaves only 52 meaningful subcarriers for further analysis.
- **Outlier Handling**: Outliers in the remaining CSI amplitude samples, which are common in real-world environments, are not removed. Instead, they are retained and incorporated into the learning process to ensure the model’s robustness.
- **Dimensionality Reduction**: For the selected 52 subcarriers, data collected over a window size *W* result in an original data dimension of 52 ∗ W. To enable faster inference, principal component analysis (PCA) is applied to reduce the input size to 3 ∗ W. These reduced-dimensional data serve as the final input to the proposed model.

As we will demonstrate in the results in Section 5.2, the reduced-dimensional data retain high accuracy. However, further reducing the dimensionality *W* beyond 3 results in significant information loss, leading to a notable decline in accuracy. It is fed into the input of the pre-trained 1D CNN model, which, in turn, determines the presence or absence of a person and returns a filtering threshold as follows:

$$\theta =\left\{\begin{array}{c} \theta^{low}\;\mathrm{in}\;\mathrm{the}\;\mathrm{presence}\;\mathrm{of}\;a\;\mathrm{person},\\ \theta^{high}\;\mathrm{in}\;\mathrm{the}\;\mathrm{absence}\;\mathrm{of}\;a\;\mathrm{person}. \end{array}\right.$$

The architecture of the proposed 1D CNN model is presented in Table 1. The input size corresponds to the configured window size *W* and *K* selected subcarriers, structured into a dimension of 3. Padding was applied to each convolution operation to maintain consistent output sizes. The rectified linear unit (ReLU) activation function was employed after each convolution layer. To mitigate overfitting, a dropout layer with a rate of 0.5 was incorporated.

The model was trained using the binary cross entropy loss function, which is well-suited for binary classification tasks, and the Adam optimizer. Training was performed for a maximum of 50 epochs with a batch size of 32. The initial learning rate was set to 0.01 and reduced by a factor of 10 every 10 epochs to facilitate convergence.

The dataset for training was collected using the auto-labeling method detailed in Section 4.2, comprising approximately 415,000 samples (10 CSI samples per second). To ensure a balanced dataset, approximately 200,000 samples were collected for each of the two classes used for training.

Recall that, as illustrated in Figure 5, our proposed framework employs a 1D CNN instead of the commonly used 2D CNN models in Wi-Fi sensing to process run-time collected CSI data. This decision allows the filtering module to function directly on-device with minimal computational overhead, which is crucial for our intended application.

The computational complexities of 1D and 2D convolutions differ substantially [29]. Specifically, convolving an image of dimensions N×N with a K×K kernel in a 2D CNN results in a computational complexity of $O(N^{2}K^{2})$, whereas the corresponding 1D convolution (with the same dimensions, *N* and *K*) has a complexity of O(NK). This implies that under equivalent conditions—such as identical configurations, network architectures, and hyperparameters—the computational complexity of a 1D CNN is significantly lower than that of a 2D CNN. Given that our goal is to develop a lightweight filtering module rather than a full-fledged classification model, the lower computational complexity of the 1D CNN makes it a highly efficient and practical solution.

## 5. System Implementation and Evaluation

### 5.1. Implementation and Experiments

In this study, the Nexmon CSI Extractor [26] for Raspberry Pi 4B bcm43455c0 was selected to implement a prototype of Wi-Filter. We deployed testbeds consisting of several Raspberry Pi transmitters and a TP-link Access Point. The CSI measurements of 52 out of 64 sub-carriers were extracted from each transmit/receiver antenna pair and stored in our server with timestamps for automatic labeling, offline processing, and model training. Wi-Filter uses a dynamic threshold mechanism with $\theta^{low}=0.3$ and $\theta^{high}=0.7$. To evaluate the proposed method, we conducted experiments using Wisenet’s four channel camera systems, SNK-B73047BW, in two classrooms. The outputs from all the systems including Raspberry Pis and cameras were timestamped from an external NTP time server for synchronization.

### 5.2. Experiment Results

The first experiment evaluates the effectiveness of the proposed auto-labeling approach and lightweight models for the real-time detection of human presence based on the use of Wi-Fi CSI. To design the lightweight component and extract the principle features sensitive to human motion in the target environments, we use principal component analysis (PCA)-based technology to reduce the CSI dimensions and select the top three principal components, that is, K=3 (in Figure 5) among 52 sub-carriers.

**Figure 6:**
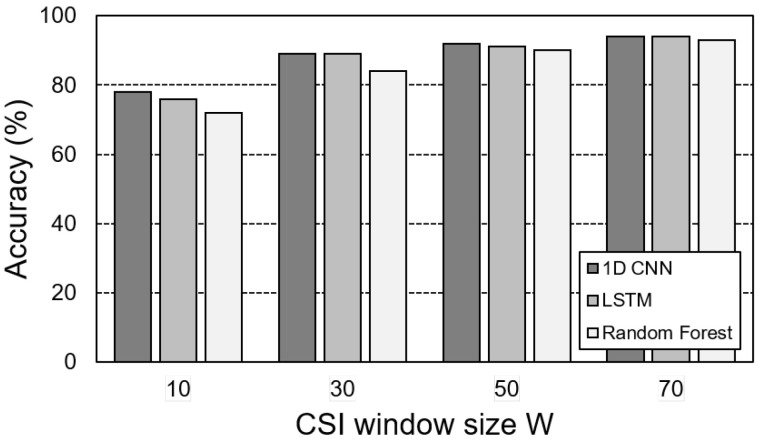
Accuracy of threshold selector for motion sensing for various window sizes.

Figure 6 compares the motion detection accuracy of three models—1D CNN, LSTM, and random forest—and examines the effect of the input size *W* on accuracy. The models were evaluated by increasing *W* in increments of 20, ranging from 10 to 70. The results indicate that all three models achieve higher accuracy as the window size increases. Based on these findings, we selected W=50, which balances accuracy and suitability for real-time classification. While both 1D CNN and LSTM demonstrated similar accuracy, 1D CNN exhibited approximately 1.3 times faster inference speed and required fewer learning parameters compared to LSTM. This significant advantage in computational efficiency, combined with its robust performance, led to the selection of 1D CNN as the optimal model for the Wi-Filter’s threshold selector.

Table 2 shows the real-time motion detection accuracy of the 1D CNN model trained via the proposed auto-labeling system in three indoor locations. For each of the three target (test) sites, the first row is the accuracy of the model built using samples newly collected at each location using the proposed automatic labeling system, and the second row is the accuracy when using the pre-trained model built on samples collected from another site. The results highlight the effectiveness of the proposed auto-labeling system, achieving high accuracy ranging from 97.2% to 98.4%.

**Figure 7:**
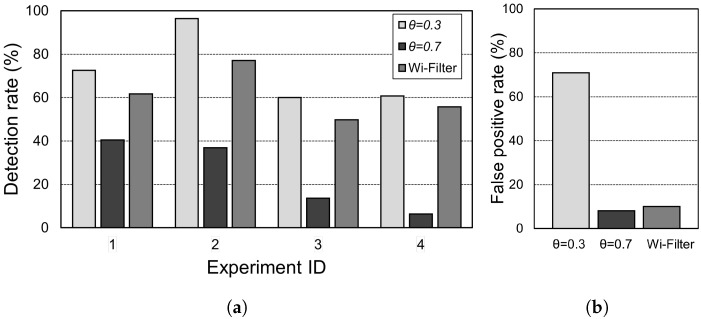
Comparison of the filtering performances of the static threshold technique (two settings) and Wi-Filter. (**a**) True positives in four different places under bright (experiment IDs 1 and 2) and dark conditions (IDs 3 and 4); (**b**) false positive.

Figure 7 presents a comparison of the filtering performances of the static threshold technique (two settings) and Wi-Filter. Experiments were performed in two locations under light (experiment IDs 1 and 2) and dark conditions (IDs 3 and 4). As depicted in Figure 7a, a low threshold value θ=0.3 indicates a relatively high detection performance. In contrast, high thresholds, particularly in dark environments, lead to very poor detection performance, causing false filtering. However, as indicated in Figure 7b, the lower the threshold, the higher the false positive value; therefore, the benefits of filtering are lost. In contrast, the proposed Wi-Filter demonstrates a very low probability of a false positive while showing a detection rate almost equal to a low threshold value.

**Figure 8:**
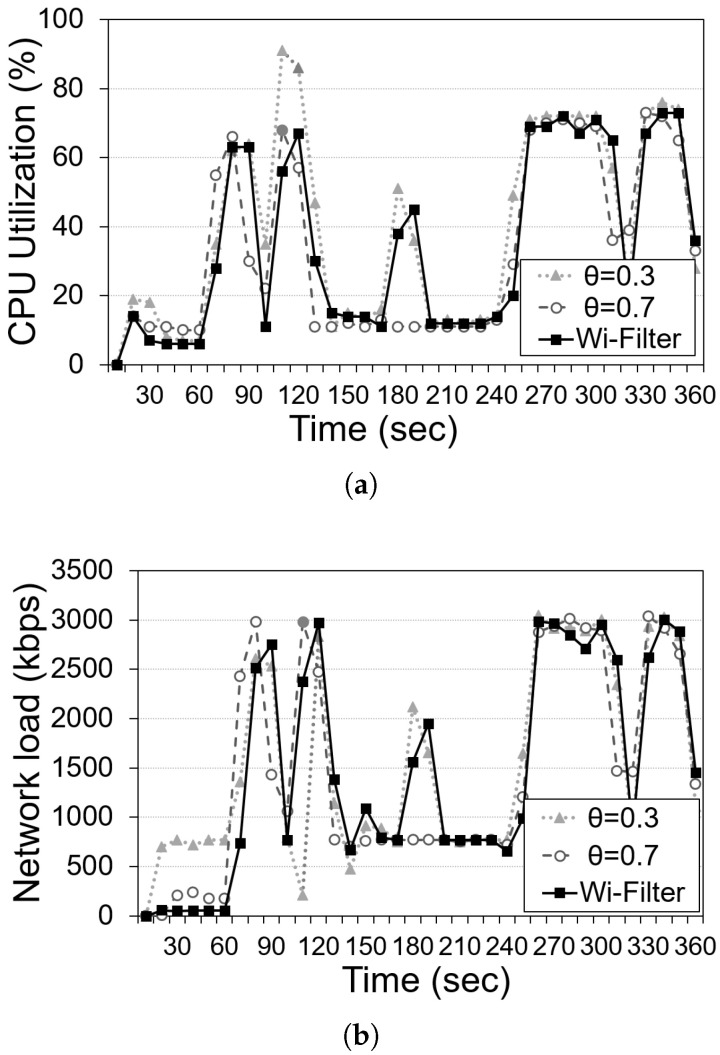
Average computing resources of the static threshold technique (two settings) and Wi-Filter: (**a**) CPU utilization, and (**b**) network transmission rate.

As an additional experiment, we measured the run-time resource usage during the real-time analysis of a selected 360 s scenario. Figure 8 depicts a comparison of the computing resource usage of the static threshold technique (two settings) and Wi-Filter for one selected real scenario: (a) CPU utilization (%), and (b) average network transmission rate (Kbps). As in the previous result in Figure 7, Wi-Filter shows a balanced resource usage between a low threshold value θ=0.3 and a high threshold value θ=0.7.

## 6. Conclusions and Discussion

In this study, we introduced Wi-Filter, a novel frame-filtering approach that opportunistically leverages Wi-Fi signals to enhance motion detection-based frame filtering. The core of Wi-Filter is a shallow-layered, 1D CNN-based Wi-Fi motion sensing module, which achieves an accuracy of up to 97.2%. This module dynamically adjusts filtering thresholds for target areas, ensuring robust motion detection performance. As a result, unnecessary frames are effectively filtered, reducing the false positive rate by over 60% compared to environments configured with lower confidence score thresholds.

Wi-Filter incorporates a self-supervised learning mechanism, enabling it to adapt to changes in target areas. Moreover, the trained model is reusable across different camera systems, offering flexibility and scalability. Beyond simple binary classification, Wi-Filter can also be extended to multi-class classification tasks by integrating deeper model architectures.

However, the system faces challenges due to the sensitivity of Wi-Fi CSI data to environmental conditions. A model trained in one target area experiences a significant drop in accuracy when applied to a different target area. Additionally, while Wi-Filter effectively addresses many issues associated with static filtering thresholds, its overall accuracy remains dependent on the reliability of the vision-based motion detector.

In our future work, we aim to enhance the adaptability and transferability of Wi-Filter through the following approaches:

(i) optimizing threshold selection through the development of dynamic mechanisms that adjust filtering thresholds based on real-time environmental and contextual factors;
(ii) integrating advanced Wi-Fi-based positioning models to make the system more versatile and applicable across diverse environments;
(iii) leveraging meta-learning techniques to enable quick adaptation to new domains with minimal additional data, thereby improving the system’s robustness and performance in dynamic scenarios.

## Figures and Tables

**Table 1:** Network architecture.

Layer	Type	Filter/Unit	Kernel Size	Output Shape
1	Input	-	-	(W,3)
2	Conv1D	128	3	(W−2,128)
3	MaxPooling1D	-	2	$\left(\frac{W-2}{2},128\right)$
4	Conv1D	128	1	$\left(\frac{W-2}{2},128\right)$
5	GlobalMaxPooling1D	-	-	(128)
6	FC	128	-	(128)
7	Dropout	0.5	-	(128)
8	FC	64	-	(64)
9	FC	1	-	(1)

**Table 2:** Accuracy of auto-labeling for three places.

Data Collection Site	1	2	3
Target site	98.1	97.2	98.4
Different site	42.8	43.3	42.2

## Data Availability

Data are contained within the article.
